# Supplementary material for: Falsifying computational models of endothelial cell network formation through quantitative comparison with in vitro models
Source: PLoS Comput Biol. 2025 Apr 30;21(4):e1012965. doi: 10.1371/journal.pcbi.1012965 (PMC12074657; doi:10.1371/journal.pcbi.1012965)
Supplement: S1 Fig — A) A detailed scheme of the image analysis pipeline showing intermediate images. B) A comparison between lacunae area measured by independent peers and automatically segmented lacunae. (PDF) [file pcbi.1012965.s001.pdf]

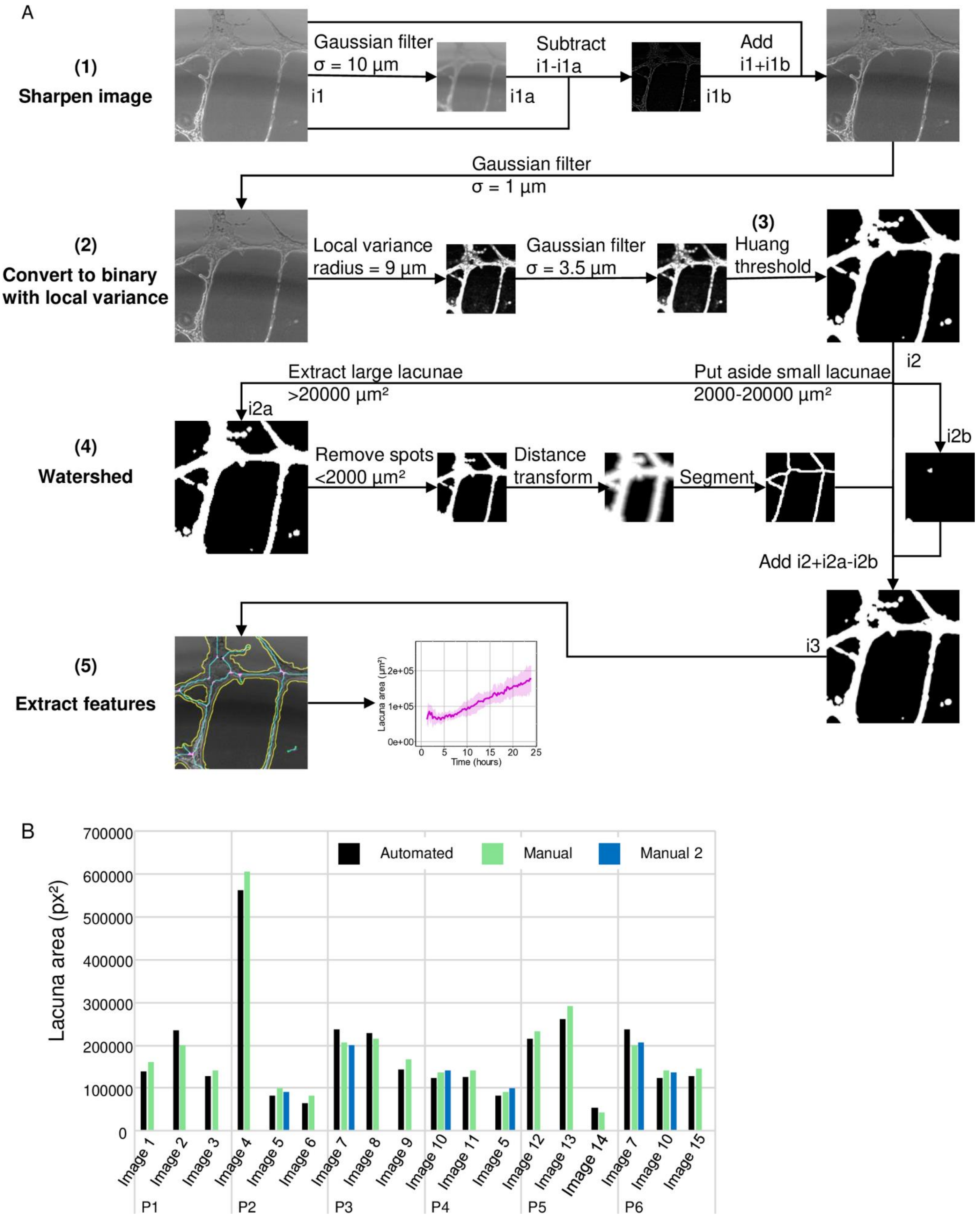

S1 Fig. The image analysis pipeline uses automatic segmentation to avoid bias in time-lapse analysis A) A detailed scheme of the image analysis pipeline showing intermediate images. B) A comparison between lacunae area measured by independent peers and automatically segmented lacunae.
