# Supplementary material for: Falsifying computational models of endothelial cell network formation through quantitative comparison with in vitro models
Source: PLoS Comput Biol. 2025 Apr 30;21(4):e1012965. doi: 10.1371/journal.pcbi.1012965 (PMC12074657; doi:10.1371/journal.pcbi.1012965)
Supplement: S2 Fig — A) ECs were labelled using membrane linker dyes and segmented from the background. B) The average area of fluorescently labelled ECs over 24 hours. (n > 600 per time step) C) The average cell length of elongated cells over time (Area between 500 and 1500 µm²; roundness below 0.25) (n > 6 per time step). D) A comparison of the number of branches per mm² for chemoattractant diffusion lengths ranging from 10 µm to 100 µm for the cell elongation model and the contact inhibition model after 2880 MCS. In magenta the in vitro number of branches per mm² after 24 hours. E) Cell covered area was measured as a percentage of the total well area. Shaded areas represent the standard deviation. F) ECs (white) in the mechanical model exert strain on their environment indicated by blue green heatmap. G) Histogram shows the relative frequency of lacuna areas in the computational models of endothelial cell network formation after 24 hours (2880 MCS) (D=5.0·10−13m2s−1;∈=1.02·10−4s−1;α=1·10−3s−1;n=8). (PDF) [file pcbi.1012965.s002.pdf]

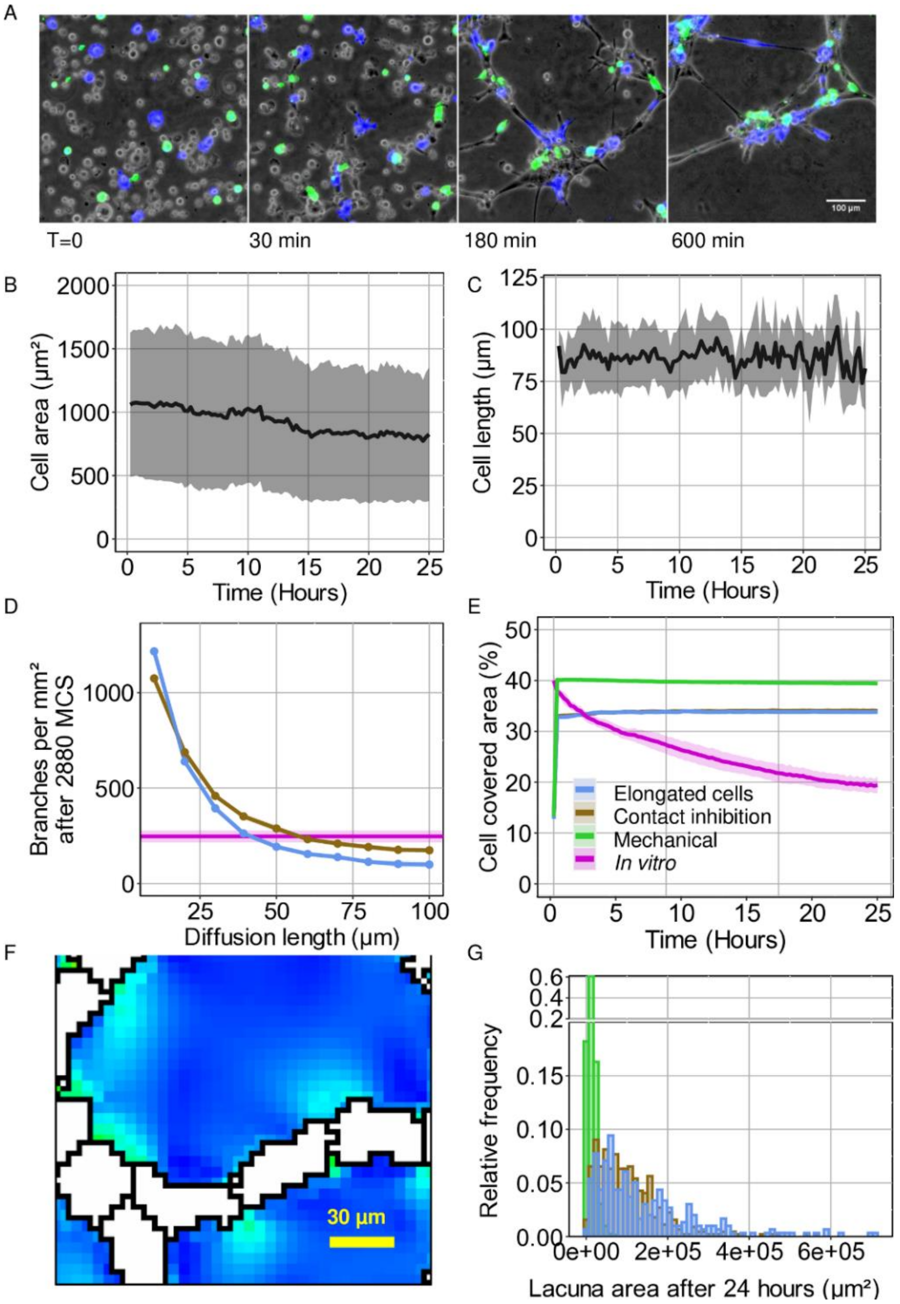

S2 Fig. Additional images and graphs A) ECs were labelled using membrane linker dyes and segmented from the background. B) The average area of fluorescently labelled ECs over 24 hours. (n > 600 per timestep) C) The average cell length of elongated cells over time (Area between 500 and 1500 µm²; roundness below 0.25) (n > 6 per timestep). D) A comparison of the number of branches per mm² for chemoattractant diffusion lengths ranging from 10 µm to 100 µm for the cell elongation model and the contact inhibition model after 2880 MCS. In magenta the *in vitro* number of branches per mm² after 24 hours. E) Cell covered area was measured as a percentage of the total well area. Shaded areas represent the standard deviation. F) ECs (white) in the mechanical model exert strain on their environment indicated by blue green heatmap. G) Histogram shows the relative frequency of lacuna areas in the computational models of endothelial network formation after 24 hours (2880 MCS) ($D = 5.0 \cdot 10^{-13} m^2 s^{-1}; \epsilon = 1.02 \cdot 10^{-4} s^{-1}; \alpha = 1 \cdot 10^{-3} s^{-1}; n = 8$).
