# Supplementary material for: Falsifying computational models of endothelial cell network formation through quantitative comparison with in vitro models
Source: PLoS Comput Biol. 2025 Apr 30;21(4):e1012965. doi: 10.1371/journal.pcbi.1012965 (PMC12074657; doi:10.1371/journal.pcbi.1012965)
Supplement: S3 Fig — A) Cell elongation model. B) Contact inhibition model. C) Mechanical model. (PDF) [file pcbi.1012965.s003.pdf]

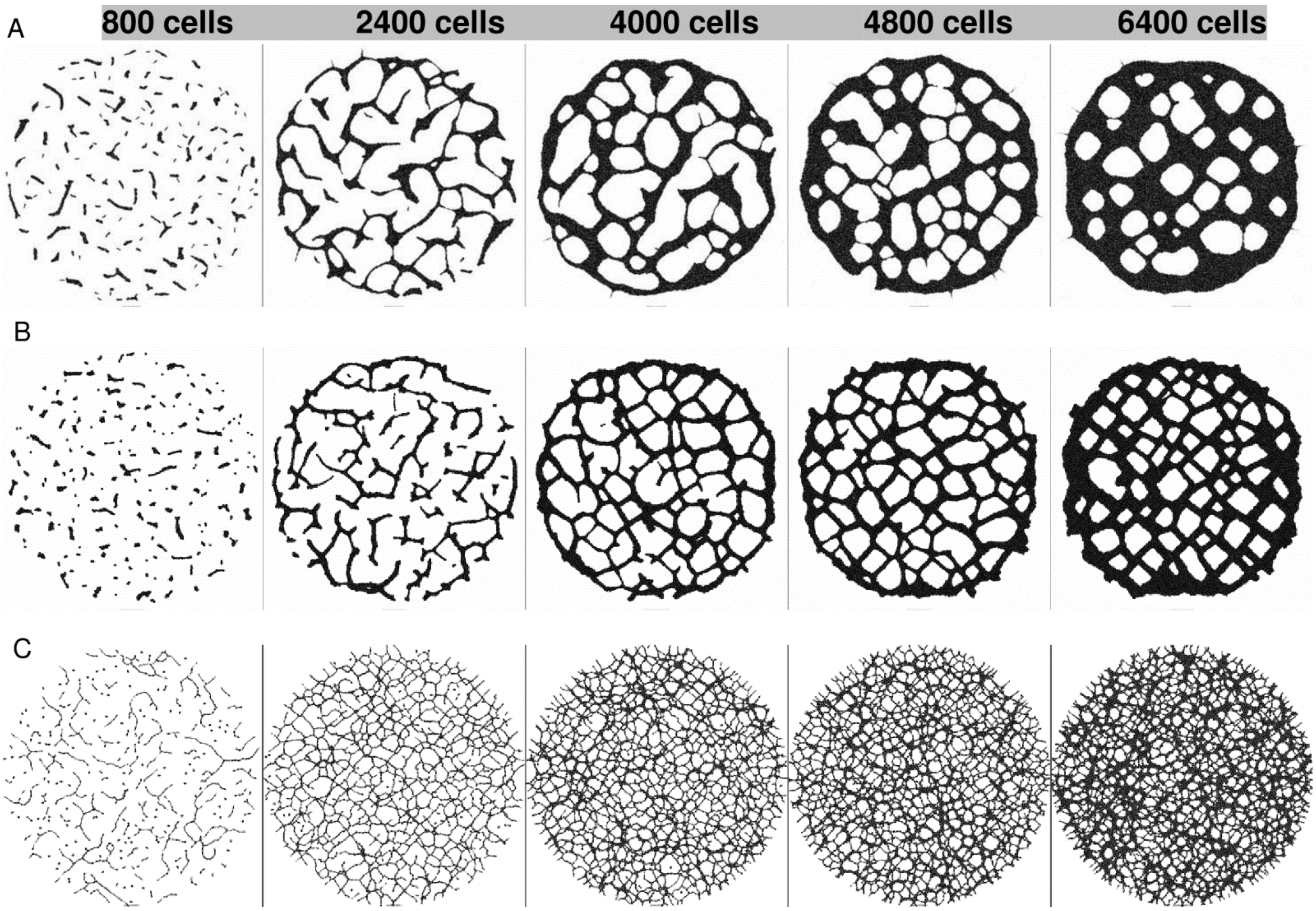

S3 Fig. Overview images of simulated networks for different cell densities after 2880 MCS. A) Cell elongation model. B) Contact inhibition model. C) Mechanical model.
