# Supplementary material for: Falsifying computational models of endothelial cell network formation through quantitative comparison with in vitro models
Source: PLoS Comput Biol. 2025 Apr 30;21(4):e1012965. doi: 10.1371/journal.pcbi.1012965 (PMC12074657; doi:10.1371/journal.pcbi.1012965)
Supplement: S4 Fig — A-D) Cell elongation model. E-H) Contact inhibition model. I-L) Mechanical model. Overview images are 4000 cells after 2880 MCS. (PDF) [file pcbi.1012965.s004.pdf]

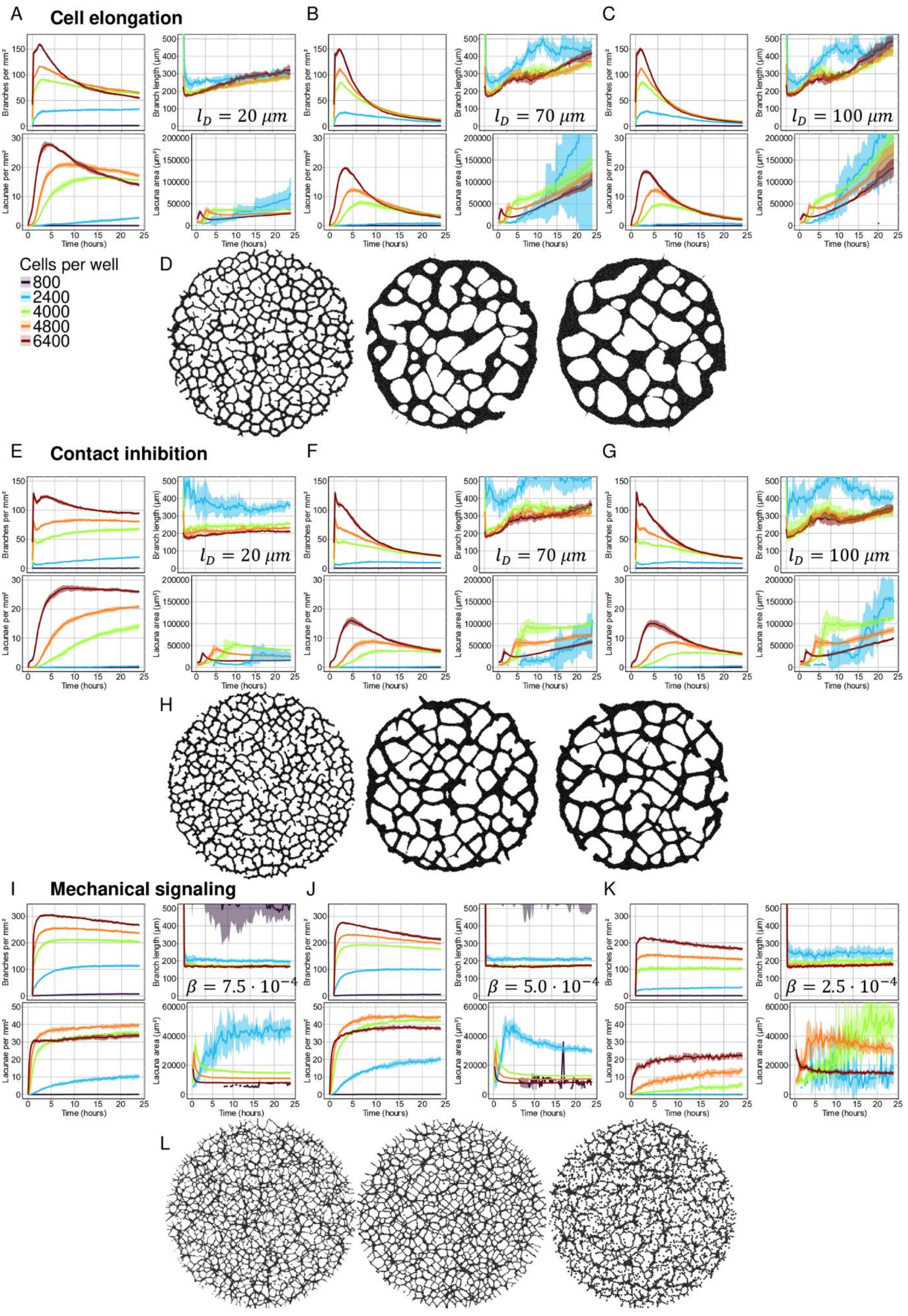

S4 Fig. Overview of dynamical analysis of simulated networks for different cell densities and diffusion length or stiffness sensitivity. A-D) Cell elongation model. E-H) Contact inhibition model. I-L) Mechanical model. Overview images are 4000 cells after 2880 MCS.
