# Supplementary material for: Falsifying computational models of endothelial cell network formation through quantitative comparison with in vitro models
Source: PLoS Comput Biol. 2025 Apr 30;21(4):e1012965. doi: 10.1371/journal.pcbi.1012965 (PMC12074657; doi:10.1371/journal.pcbi.1012965)
Supplement: S5 Fig — A) HMEC-1 cells were seeded at different densities. B) 10 mM l-lactate was added to the MCDB-131 medium prior to the start of the tube formation assay. C) HMEC-1 cells were seeded on either growth factor reduced Matrigel or regular Matrigel. HCl was added to the medium prior to the start of the tube formation assay to reduce its pH to 6.0. D) Fetal calf serum was added to the medium in different percentages. E) HMEC-1 cells were seeded on either growth factor reduced Matrigel or regular Matrigel. VEGF-A was added to the medium. F) HMEC-1 cells were seeded on either regular Matrigel diluted in DMEM high-glucose. (PDF) [file pcbi.1012965.s005.pdf]

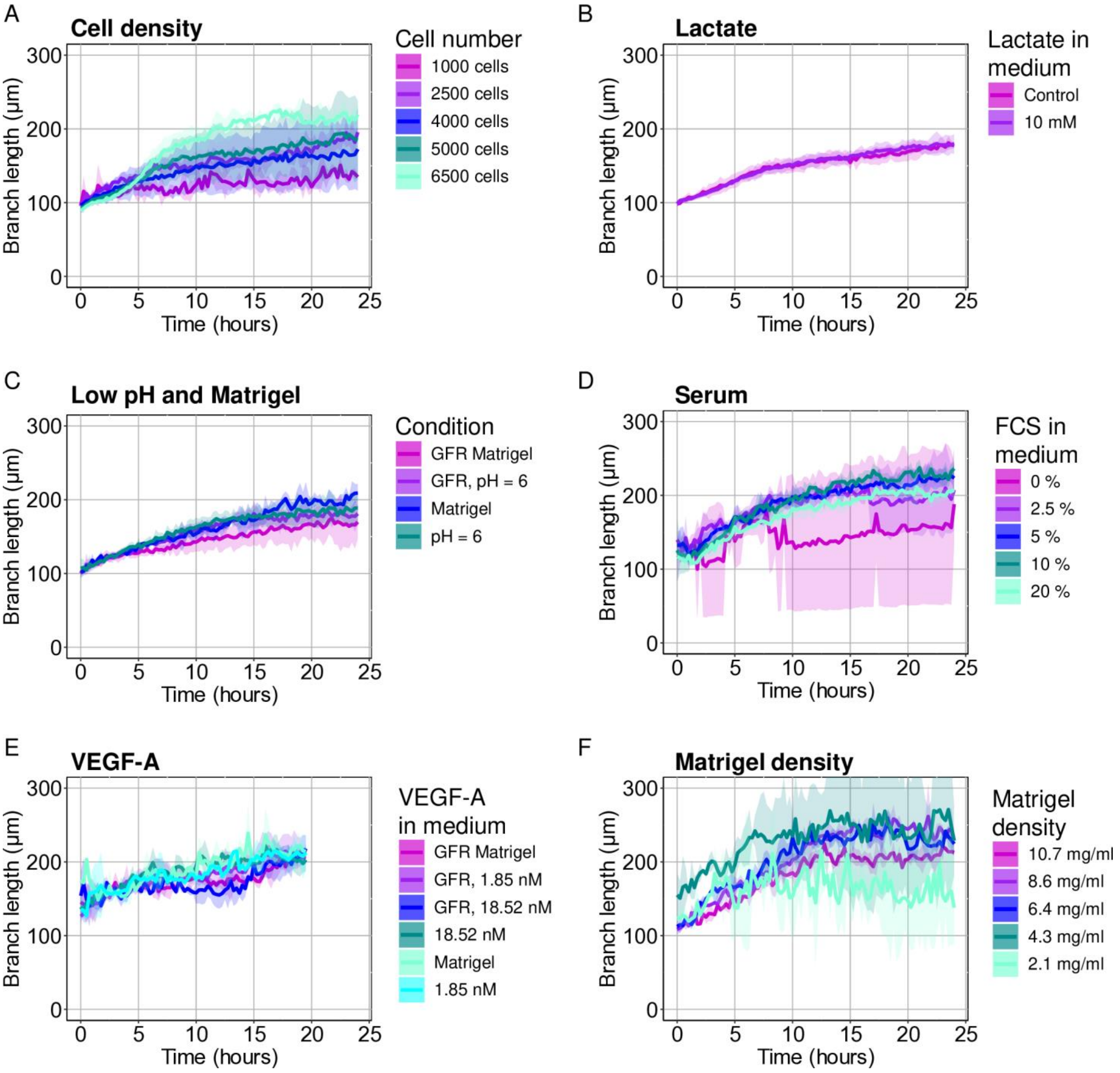

S5 Fig. Overview of branch length analysis of *in vitro* endothelial cell networks for different experimental conditions. A) HMEC-1 cells were seeded at different densities. B) 10 mM l-lactate was added to the MCDB-131 medium prior to the start of the tube formation assay. C) HMEC-1 cells were seeded on either growth factor reduced Matrigel or regular Matrigel. HCl was added to the medium prior to the start of the tube formation assay to reduce its pH to 6.0. D) Fetal calf serum was added to the medium in different percentages. E) HMEC-1 cells were seeded on either growth factor reduced Matrigel or regular Matrigel. VEGF-A was added to the medium. F) HMEC-1 cells were seeded on either regular Matrigel diluted in DMEM high-glucose.
