# Supplementary material for: Falsifying computational models of endothelial cell network formation through quantitative comparison with in vitro models
Source: PLoS Comput Biol. 2025 Apr 30;21(4):e1012965. doi: 10.1371/journal.pcbi.1012965 (PMC12074657; doi:10.1371/journal.pcbi.1012965)
Supplement: S1 Table — (PDF) [file pcbi.1012965.s006.pdf]

S1 Table. Chemotaxis model parameters

Parameter	Value	Unit
Pixel size	2e-6	m
Field diameter	1900	pixels
CPM temperature	50	-
Cell target area	250	pixels
Initial cell diameter	12	pixels
Strength of area constraint	50	-
Strength of length constraint	5	-
Connectivity dissipation	5000	N m^{-1}
Cell-medium contact cost	20	-
Cell-cell contact cost	40	-
Chemotaxis strength	1000	N m^{-1}
Relaxation time	100	MCS
Border energy	100	N m^{-1}
Order of neighbourhood	2	-
Diffusion coefficient	5e-13	m^2 s^{-1}
Secretion rate	1e-3	s^{-1}
PDE time increment	2	s
PDE iterations per MCS	15	-
