# Supplementary material for: Falsifying computational models of endothelial cell network formation through quantitative comparison with in vitro models
Source: PLoS Comput Biol. 2025 Apr 30;21(4):e1012965. doi: 10.1371/journal.pcbi.1012965 (PMC12074657; doi:10.1371/journal.pcbi.1012965)
Supplement: S2 Table — (PDF) [file pcbi.1012965.s007.pdf]

S2 Table. Mechanical model parameters

Parameter	Value	Unit
Pixel size	0.000005	m
Field diameter	760	pixels
CPM temperature	1	-
Cell target area	40	pixels
Strength of area constraint	10000	-
Cell force	1	$N\ m^{-1}$
Cell-medium contact cost	500000	m^{-1}
Cell-cell contact cost	1000000	m^{-1}
Young's modulus	12000	Pa
Poisson's ratio	0.45	-
Accuracy of solver	0.00001	-
Strength of durotaxis	24	-
Threshold for stiffness preference	15000	Pa
Strain stiffening parameter	0.1	-
