# Supplementary material for: Falsifying computational models of endothelial cell network formation through quantitative comparison with in vitro models
Source: PLoS Comput Biol. 2025 Apr 30;21(4):e1012965. doi: 10.1371/journal.pcbi.1012965 (PMC12074657; doi:10.1371/journal.pcbi.1012965)
Supplement: Attachment — Submitted filename: Response to reviewers 1.pdf [file pcbi.1012965.s013.pdf]

Ir. Tessa Vergroesen and Prof. dr. Roeland Merks
Mathematical Institute and Institute of Biology Leiden
Leiden University, Einsteinweg 55, 2333 CC Leiden
Tel. +31-6-45231485
E-mail: merksrmh@math.leidenuniv.nl
Date: 16/01/2025

Dear Editor,

Thank you for the opportunity to revise and resubmit our manuscript titled "*Falsifying computational models of angiogenesis through quantitative comparison with in vitro models*" (Manuscript ID: PCOMPBIOL-D-24-01420). We would like to express our sincere appreciation to the reviewers for their time and thoughtful comments. We believe that their feedback has significantly improved the quality of our work. Below, we provide a summarised response to the reviewers followed by a point-by-point response to each of the reviewers' comments.

In response to Reviewer #1's concerns, we have ensured consistent use of the term "endothelial cell network formation" throughout the manuscript, replacing "angiogenesis" to better reflect the biological context. Additionally, we have clarified our rationale for selecting the model parameters, now explicitly referencing previous studies and sensitivity analyses to provide a clearer foundation for our choices. In response to Reviewer #2's feedback, we refined the image analysis pipeline to better represent the early stages of network formation. This includes adjustments to the segmentation process, which have improved the representation of lacuna dynamics. We also expanded our analysis of model behaviour by incorporating additional metrics such as lacuna area dynamics and more comprehensive time-series data. Furthermore, we have toned down the discussion regarding the explanatory power of the cell elongation model, acknowledging its limitations and providing a more cautious interpretation of our findings. Finally, in response to Reviewer #3, we have made the image analysis code publicly available on GitHub, along with a detailed readme for ease of use. We also addressed the biological mechanisms underlying the effect of cell density in our experiments, offering more insight into the factors that may influence network formation. Lastly, we expanded the discussion on the limitations of 2D models and the potential advantages of shifting to 3D models, considering recent advancements in computational power and their relevance for more complex *in vitro* and *in silico* angiogenesis studies.

Point-by-point response:

Reviewer #1:

- This paper deals with an in vitro assay and in silico simulations where disperse endothelial cells form a capillary network de novo, followed by a network

remodelling phase. It is very confusing to call that process angiogenesis as it is widely accepted that it consist in the formation of new vessels from pre-existing ones, and obviously there are no existing vessels here. I am sure the authors know that very well, so I can't understand their rational of using the term angiogenesis. This should be replaced throughout the manuscript by a more appropriated term such as "de novo vascular development" or "vasculogenesis".

*We agree with Reviewer #1's feedback regarding the use of the term angiogenesis. In response, we have ensured the consistent use of the term 'endothelial cell network formation' in the revised version of the manuscript. We have decided on endothelial cell network formation and not vasculogenesis, since in vasculogenesis a primitive network forms from endothelial precursor cells which de novo differentiate into endothelial cells. We also added the following to the first paragraph of the introduction to clarify this difference further in support of our choice of words: "*During embryogenesis, endothelial precursor cells, angioblasts, migrate and differentiate to form the first blood vessels. This process of blood vessel formation by *de novo* endothelial cell (EC) production, is called vasculogenesis [1]. After vasculogenesis, the primary vascular network is expanded by angiogenesis.*"*

- The authors do not use formal parameter inference, for instance using approximate Bayesian computation like in Alamoudi et al Bioinformatics 2023. Instead, to "match" the properties of the in silico networks generated by the chemotaxis models with the ones generated in vitro, they fixed all parameters except one that is varied until observing similarities. In the mechanical model, all parameters are fixed and variations were not explored in this paper. This is a poor approach that seems like cherry picking without a convincing explanation. It is possible that there are other parameter regimes that have a better fit to the experimental situation. This is not explored in depth and should be done properly or at least clearly mentioned.

We agree that the manuscript would benefit from a more thorough and more formal parameter exploration. In this manuscript we specifically focused on comparing existing models to in vitro *experiments in a high-throughput manner and rely in parts on parameter regimes that have been found in earlier global sensitivity analysis, based on the Sobol' method on the contact inhibition model (Boas* et al. *BMC Syst Biol. 2015). To clarify the choice of parameters further, we have added the following sentence to the introduction:* "We select model parameters from the original work [14,27,28] and from published global sensitivity analyses [32], then we fit the models to our own *in vitro* observations, and finally we present additional parameter examinations." *and to the discussion:* "To compare the models to our *in vitro* time-lapses we selected parameters from the original work [14,27,28] and from published sensitivity analyses [39], and fitted parameters to our own *in vitro* observations (S2 Figs A-C)." *In future work we plan to use novel higher-throughput methods of parameter inferences such as those presented in*

Alamoudi et al. *(2023) in combination with our image analysis pipeline to expand on our current comparison of models to* in vitro *output. With regards to the mechanical model we found this model to be extremely sensitive to changes in parameters with regards to its capability of producing cellular networks. We did not find a parameter regime in which we were able to change the model outcomes to appear more like later stages of the* in vitro *experiments quantitatively. To state this more clearly, we have added the following to the discussion section of the manuscript:* "For the mechanical cell-cell communication model we were not able to find such a parameter regime. The mechanical model was very sensitive and often lost its ability to form networks after small changes in the explored parameters. Its high computational cost also made it difficult to perform large scale parameter sweeps." *and* "To find new parameter regimes in the mechanical model we could combine our presented image-analysis pipeline with topological data analysis [44] and novel high-throughput parameter inference methods such as FitMultiCell [45], which combines the iteratively changing of parameters, the running of simulations and the assessment of the model outcomes in one tool, to streamline the model fitting process.*" and "*In the future we plan to expand our analysis of EC networks through more sophisticated data-analysis techniques such as topological data analysis [44] and Sobol' analysis [32] for falsifying and refining our understanding of endothelial network formation using *in vitro* and *in silico* modelling in hopes that such techniques can be generalized to the dynamics of other models of biological morphogenesis*"*

- In the experiment, there is a clear difference between the first 6 hours (aggregation, spreading and elongation) and the following hours (network remodelling). According to figure 2, none of the models seem to reproduce any of the network features in the first period, at least in the explored parameter regimes. This should be more clearly stated in the manuscript. In particular, reference 14 shows how cells exhibit significant displacement before starting to elongate. This cannot be reproduced by the elongation model where cells artificially elongate very early due to the elongation constraint.

We agree that we had not yet addressed the large difference in the earliest stage of network formation in the manuscript. It is true that the cell elongation does not reproduce the early stages of endothelial network formation by elongating before displacing. The cell elongation model explores the ability of cell elongation as a driving force behind cell network formation and sprouting and therefore begins with cell elongation, before chemotaxis. This is a feature of the model and we can indeed conclude that it does not represent in vitro *cell behavior well. We have added the following to the discussion to state this more clearly:* "There were also clear discrepancies between the cell elongation model and *in vitro* network formation. For example, Parsa *et al.* (2011) observed that in vitro ECs aggregate and spread first, before elongating [15]. Thus, *in vitro*, the earliest stages of network formation seem not driven by elongation, as assumed by the cell elongation model"

- In the abstract, it is mentioned that the response of silico and in vitro networks are studied with respect to perturbations of key model parameters and associated experimental ones. This is a crucial aspect to investigate the validity of model mechanisms. However, in this paper this is only done for the initial cell density. Although this could be seen as a model parameter, it is not really related to the model mechanisms that are investigated, like elongation or the autocrine chemotaxis mechanisms. If the authors are not planning to investigate other key parameters, the abstract sentence mentioned above should be reformulated by a much more explicit one, mentioning the cell density variation. Also in the discussion the second paragraph should be toned down, as no "assess if systematic, concurrent parameters changes in vitro and in silico" was really done.

We have changed the sentence in the abstract to "We test the response of the *in silico* dynamical cell network characteristics to changes in cell density and make related changes in the *in vitro* experiments." *We also included an additional supplementary Fig 5 showing the* in vitro *effect on branch length of the presence of VEGF-A, lactate, HCl , growth factors or fetal calf serum in the medium and a dilution of Matrigel on endothelial network formation. Since the cell density had the most significant effect on the* in vitro *outcome, we chose this parameter for the* in silico *to* in vitro *comparison as we are looking for domains where the* in silico *models stop reproducing the* in vitro *experiments. We changed the sentence in the discussion to* "Within the selected parameter regimes, we compared the response of the *in silico* models and the *in vitro* networks to changes in cell density to further assess the correctness of the models (Figs 3 and 4)."

- The final sentence in the abstract is also overstated: "...our dynamic approach helps to clarify key endothelial cell interactions required for angiogenesis, and how the approach helps analyze what key changes in network properties can be traced back to changes in individual cell". In fact, based on the comparison made, all three models can be rejected. What is the mechanism in the three models that is required for angiogenesis? It is very difficult to say something about an isolated mechanisms if that mechanisms cannot be perturbed in the in vitro and in silico cases simultaneously. I think the only thing we learn is that the combination of mechanisms (and their corresponding modeling choices) present in the three analysed models are not correct.

The last sentence of the abstract was revised to "Of the three computational models that we have considered, the cell elongation model best captures the remodelling phase of *in vitro* endothelial cell network formation. Furthermore, in the *in vitro* model, the final size and number of lacunae in the network are independent of the initial cell density. This observation is also reproduced in the cell elongation model, but not in the

other two models that we have considered. Altogether, we present an approach to model validation based on comparisons of time-resolved data and variations of model conditions."

- For the sake of reproducibility and correctness, experimental data and code should be publicly available. Publishing the data will also allow other models with different mechanisms to be compared too. It is possible that a combination of chemical and mechanical cues are both playing important roles. That was not included in any mode tested, but has been done recently with a cellular Potts model too (Chiang et al 2024 Plos Comp Bio).

We have uploaded the image analysis pipeline to github.com/TMVergroesen/NetworkAnalysis, including sample files and readme documentation. We have made the time-lapses and simulation data available at Zenodo. Furthermore, we agree that it is a likely possibility that a combination of chemical and mechanical cues work together to guide the endothelial cells in their network formation. To state this more clearly we have added the following to the discussion section of the manuscript: "Many computational models of EC network formation, based on assumptions like cell-cell attraction [28], cell shape effects [14,23], cell-ECM interaction [21,27], or a combination of the former [38], have been successful at simulating the formation of EC networks that qualitatively resemble those observed *in vitro*."

Reviewer #2:

- The paper's central claim rests on the similarity between the in silico models and in vitro results, with Figure 2 serving as a primary dataset. However, the basis for the assertion of similarity is insufficiently clear. For instance, in the case of lacuna area dynamics (Figure 2D), although the final output of the elongated cell model aligns most closely with in vitro data, the early-stage kinetics differ significantly. The manuscript discusses similarity by comparing only two time points, yet, as time-series data, the dynamics should be analyzed comprehensively over the entire timeline to capture the true behavior. Given the aim of this study—to identify an in silico model capable of explaining angiogenesis mechanisms—the evolution of network structure over time is a critical perspective that is currently underdeveloped.

The data in the initial stages, in particular those describing lacuna area, was significantly distorted by choices in the image analysis pipeline that detected large, joint lacunae in the early stages of network formation when cells were not yet connected with one another. We thank the reviewer for bringing this issue to our attention. We have adjusted the lacuna segmentation in the image analysis pipeline and updated its description in the Methods section. The earlier difference in lacunae dynamics is partially caused by

an artifact introduced by the image analysis tool due to large enclosed areas in the network being labeled as lacunae. We made a small change in the settings of the "Analyze Particles" plugin to exclude lacunae with a circularity smaller than 0.2. This excludes very elongated or twisted structures and thereby removes these larger accidentally enclosed areas from the lacunae list. We also turn off the watershed step in the image segmentation for the first hour of network formation, since we do not expect any networks to form in the first hour and the watershed can occasionally create false positive connections between dispersed cells. These two changes in the image analysis pipeline resulted in a much better representation of the network for the first 6 hours, when the network is not fully formed or percolated yet.

- Furthermore, in Figure 4, the response of in silico models to changes in initial cell number is analyzed only in terms of branch count. To gain a more thorough understanding, at least, the dynamics of lacuna area should also be assessed. Although the cell elongation model may show the closest resemblance to in vitro outcomes among the three models, the current data do not substantiate a strong explanatory power in an absolute sense, only in a relative comparison.

We have added graphs showing the dynamics of additional features, including lacuna area, alongside some example images to the supplementary material (S4 Fig).

- Employing more comprehensive quantitative metrics from graph theory could provide a richer, multidimensional analysis of network structure.

We agree with Reviewer #2 that graph theory could provide a richer analysis of the differences between the in silico *models and the* in vitro *timelapses. We will explore topological data analysis of the data presented here in future collaborative work. We have added the following to the discussion section:* "Another interesting addition to the image analysis pipeline would be the inclusion of topological data analysis (TDA) [44]. The time-lapses of the segmented branches and lacunae could be regarded as multidimensional graphs suitable for further analysis using TDA. TDA could also be expanded to work on 3D time-lapses to analyse 3D *in vitro* EC sprouting assays [61,62],"

- In the Discussion, the explanatory power of the cell elongation model is attributed to factors such as long-range connections, slower movement along the short axis, and lower network density. Notably, the phrase "We can conclude…" in the third paragraph seems overconfident given the speculative nature of the current discussion. To strengthen these claims, the authors should perform additional computational experiments to directly test the impact of these specific parameters.

We have toned down the discussion on the comparison of the chemotaxis-models and changed the final part of this discussion to "Thus, the cell elongation seems more robust in reproducing the remodelling stage of *in vitro* EC network formation than the contact inhibition model."

Reviewer #3:

- The code or software should be publicly available by the authors.

We have shared the image analysis code on github.com/TMVergroesen/NetworkAnalysis *with a readme file explaining how to use the software. The code for the computational models is available through the respective primary articles.*

- While the paper describes network formation at higher cell densities both in the in vitro and in silico, it lacks the biological mechanism behind this behavior. A brief discussion on potential factors or mechanisms, such as signaling pathways or spatial constraints, would be more biologically insightful.

We have added the following to the discussion to make the discussion of the effect of cell density on the experiment more biologically insightful: "Cell density naturally affects the distance between cells right after seeding, and can therefore affect the ability of cells to sense their neighbours at the start of the experiment [5,13,15]. Cells also influence the experimental environment by digesting nutrients and excreting signalling molecules [69], but also by actively remodelling the ECM [5,70]."

- The limitations with 2D models and discussion about the feasibility and challenges toward shifting to 3D models would enhance the study.

We have added the following to the discussion to state the limits of 2D modeling more clearly, and to mention the recent developments in 3D modeling: "TDA could also be expanded to work on 3D time-lapses to analyse 3D in vitro EC sprouting assays [61,62], 3D vessels-on-a-chip sprouting assays [63] or even in vivo systems, like the developing zebrafish embryo [64]. 3D *in vitro* systems have the benefit of both having a high degree of control over the experimental environment, as allowing for 3D vascular growth and EC migration. ECs *in vivo* migrate both in 2D over the basal membrane, as in 3D during sprouting into the ECM [65]. 2D models of EC network formation can be regarded as a projection of a 3D system and adapted to study different migration types, but recent improvements in computational power have allowed the increased usage of more complex 3D models of angiogenesis [66,67]. 3D models, both *in vitro, in vivo* and *in silico* allow for a greater understanding of the complex interactions ECs have with the fibrous ECM during sprouting [68]"

Once again, we thank the reviewers for their constructive feedback. We hope that you and the reviewers will agree that the revisions we have made, as outlined above, have improved the manuscript. We look forward to hearing your thoughts on the revised version and are happy to address any further concerns.

We appreciate your consideration of our revised manuscript.

Sincerely, on behalf of the whole author team,

Tessa Vergroesen and Roeland Merks
